# In-Silico Predictions of Drug Resistance in Lung Cancers With EGFR Mutation

**DOI:** 10.1145/3732775.3733588

**Published:** 2025-06-20

**Authors:** Ibrahim Imam, Usman L. Abbas, Christian M. Gosser, Christine F. Brainson, Wibe de Jong, Xiaoqi Liu, Hunter N.B. Moseley, Shao Qing, Shulin Zhang, Ralph Zinner, Sally R. Ellingson

**Affiliations:** Chemical and Materials Engineering, University of Kentucky, Lexington, KY, USA; Chemical and Materials Engineering, University of Kentucky, Lexington, KY, USA; Toxicology and Cancer Biology, University of Kentucky, Lexington, KY, USA; Toxicology and Cancer Biology, University of Kentucky, Lexington, KY, USA; Computational Science Department, Lawrence Berkeley National, Laboratory, Berkeley, CA, USA; Toxicology and Cancer Biology, University of Kentucky, Lexington, KY, USA; Department of Molecular and Cellular, Biochemistry, University of Kentucky, Lexington, KY, USA; Chemical and Materials Engineering, University of Kentucky, Lexington, KY, USA; Pathology and Laboratory Medicine, University of Kentucky, Lexington, KY, USA; Division of Medical Oncology, University of Kentucky, Lexington, KY, USA; Division of Biomedical Informatics, University of Kentucky, Lexington, KY, USA

**Keywords:** computational drug discovery, drug resistance, high-performance computing, computational approaches in cancer, Applied computing → Life and medical sciences, Computing methodologies → Modeling and simulation

## Abstract

Cancer treatment is often hindered by the emergence of drug resistance, frequently driven by novel mutations in oncogenes or drug-targeted pathways. Predicting resistance mechanisms is critical for informing therapeutic strategies and improving patient outcomes. Here, we present a computational workflow that leverages high-performance computing (HPC) resources to systematically evaluate the impact of emerging mutations on drug efficacy. Our workflow integrates deep learning structure prediction, molecular dynamics simulations, molecular docking, and binding predictions of known compounds to predict resistance mechanisms and propose alternative therapeutic options. We also explore quantum chemical calculations as a tool to complement experimental validations to better understand the binding preferences between different protein forms.

## 1 Introduction

Drug resistance is one of the most challenging problems in cancer therapy. Drug resistance can occur when a mutation changes the target protein in cancer cells, weakening the interactions with drugs. Drug resistance is one of the leading causes of relapse in cancer and is responsible for up to 90% of cancer-related deaths[28]. An estimated 30%–55% of non-small cell lung cancer (NSCLC) patients have a relapse, resulting in death [26, 28]. NSCLC is the most prevalent form of lung cancer and is the leading cause of cancer deaths. Kentucky has the highest age-adjusted annual incidence and death rates of lung and bronchus cancers in the United States. While drug resistance information is known for common mutations related to FDA-approved drugs, due to the complexity of genomic changes in NSCLC individual patients may often harbor novel mutations in which the best treatment strategy is not known. It is imperative to have reliable methods to quickly assess the clinical relevance (resistance causing with potential for alternative treatments) of novel mutations to make personalized treatment plans and ultimately improve the quality of care and life expectancy in NSCLC patients.

Activating mutations in the Epidermal Growth Factor Receptor (EGFR) are present in 30% of NSCLC [10], and treatment of these tumors with EGFR tyrosine kinase inhibitors (TKIs) is one of the best examples of precision medicine for cancer treatment. However, resistance to TKIs remains a clinical issue. Patients receiving first- or second-generation EGFR tyrosine kinase inhibitors predominantly develop EGFR-dependent resistance, whereas about 20% of patients receiving the third-generation drug osimertinib as second-line therapy reveal on-target resistance mechanisms [19, 23]. Current data predict that 10–15% of patients develop EGFR-dependent resistance when treated with first-line osimertinib [22]. However, there are a large percentage of patients for which resistance mechanisms are unknown. Uncommon EGFR mutants may contribute to EGFR TKI therapy resistance [18, 20]. However, to evaluate these mutations in a timely manner, we must develop ways to model and understand the effects of the mutations on EGFR structure, function, and drug resistance.

One challenge in treating novel mutation-induced therapy resistance is the lack of efficient methods to find personalized treatment options. A major reason is the lack of a three-dimensional (3D) structure of uncommon mutants [20]. While common mutants of EGFR have been reported, more uncommon mutants are yet to be studied, even though they have been reported in clinical tests because of the advancement in genomic profiling of cancers. For patients with uncommon mutations, traditional treatments may lose effectiveness due to the conformational variation induced by the new mutation. To advance the treatment of lung cancer related to uncommon mutants, reliable computational prediction of protein structures may provide an alternative approach since the current techniques to experimentally determine a therapeutic target structure are impractically slow.

Artificial intelligence (AI)-based protein 3D structure prediction toolkits such as AlphaFold [4, 13] enable researchers to obtain any protein structure using the amino acid sequence. The impressive score of AlphaFold2 on Critical Assessment of Protein Structure Prediction (CASP) inspired us to explore if an AlphaFold2 predicted structure is accurate enough for a computational workflow to predict the potential for a mutation to lead to drug resistance. AlphaFold2 can shed light on the local effects of missense mutations [14] and molecular dynamics (MD) simulations can be used to refine and reveal the mutation-induced conformational variation of proteins in solution [11, 24].

While some studies have demonstrated the potential of AlphaFold2 in capturing local structural perturbations caused by mutations, others have highlighted its limitations [7, 8, 16]. Systematic discrepancies in AlphaFold2’s predictive reliability have been reported, with variations observed across different amino acid types, secondary structures, and even protein sizes. Studies have shown that AlphaFold2 performs better for medium-sized proteins compared to smaller or larger ones, likely due to biases inherent in its training data and model architecture [2]. To account for these limitations, we incorporate molecular dynamics (MD) simulations to refine the predicted structures and capture the dynamic conformational changes induced by mutations. This hybrid approach enhances our ability to assess the structural impact of mutations in the context of drug resistance while addressing potential biases in AlphaFold2’s structural predictions. In this study, we use an ensemble of structures generated by AlphaFold2 followed by MD as input for in-silico ligand screening.

**Figure 1:**
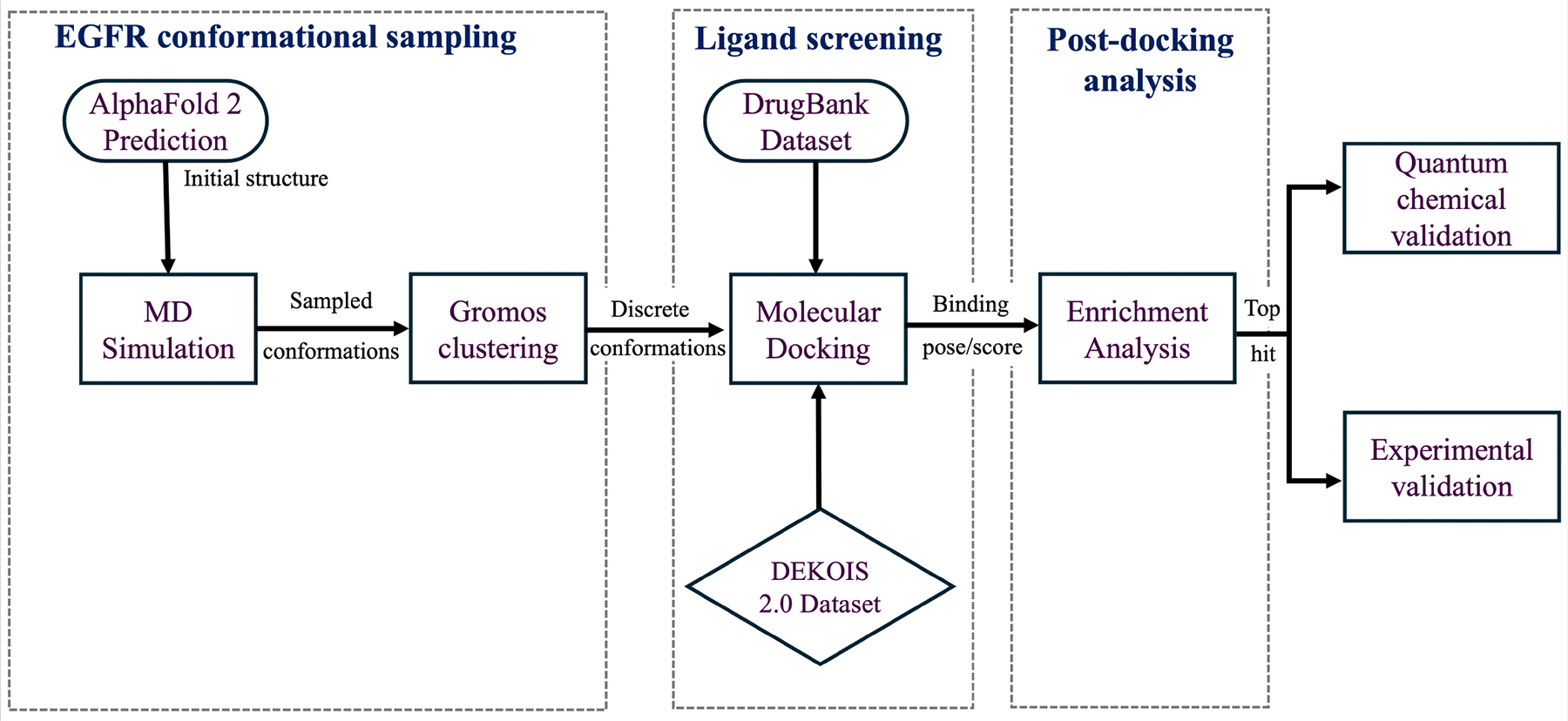
Schematic representation of computational tools to experimental validation.

In-silico ligand screening identifies small molecules that have the potential to bind to and regulate protein activities. Available in-silico screening methods range from molecular docking, which searches possible ligand conformations in a binding site using a simple scoring function, to free energy calculations, which are theoretically accurate but sensitive to the input data and computationally expensive. These methods construct a workflow (Figure 1) that identifies potential therapeutics from FDA-approved small-molecule drugs. Screening known binding compounds for a protein can also provide insight into the potential of a mutation to cause drug resistance.

For this work, we used the wild-type (WT) sequence and the sequences of three mutations. L858R and del19 are known cancer driver mutations, and T790M is a secondary mutation that causes resistance to previous first-line treatments. Osimertinib was developed for patients with T790M mutation and has become a first-line treatment. The data presented here uses T790M as an example due to the number of experimental structures for comparison. However, future work will not focus on this mutation as using osimertinib as a first-line treatment has decreased this mutation from the clinical population.

## 2 Results

### 2.1 The ability of AlphaFold2 to reproduce structures of known point mutations

**Figure 2:**
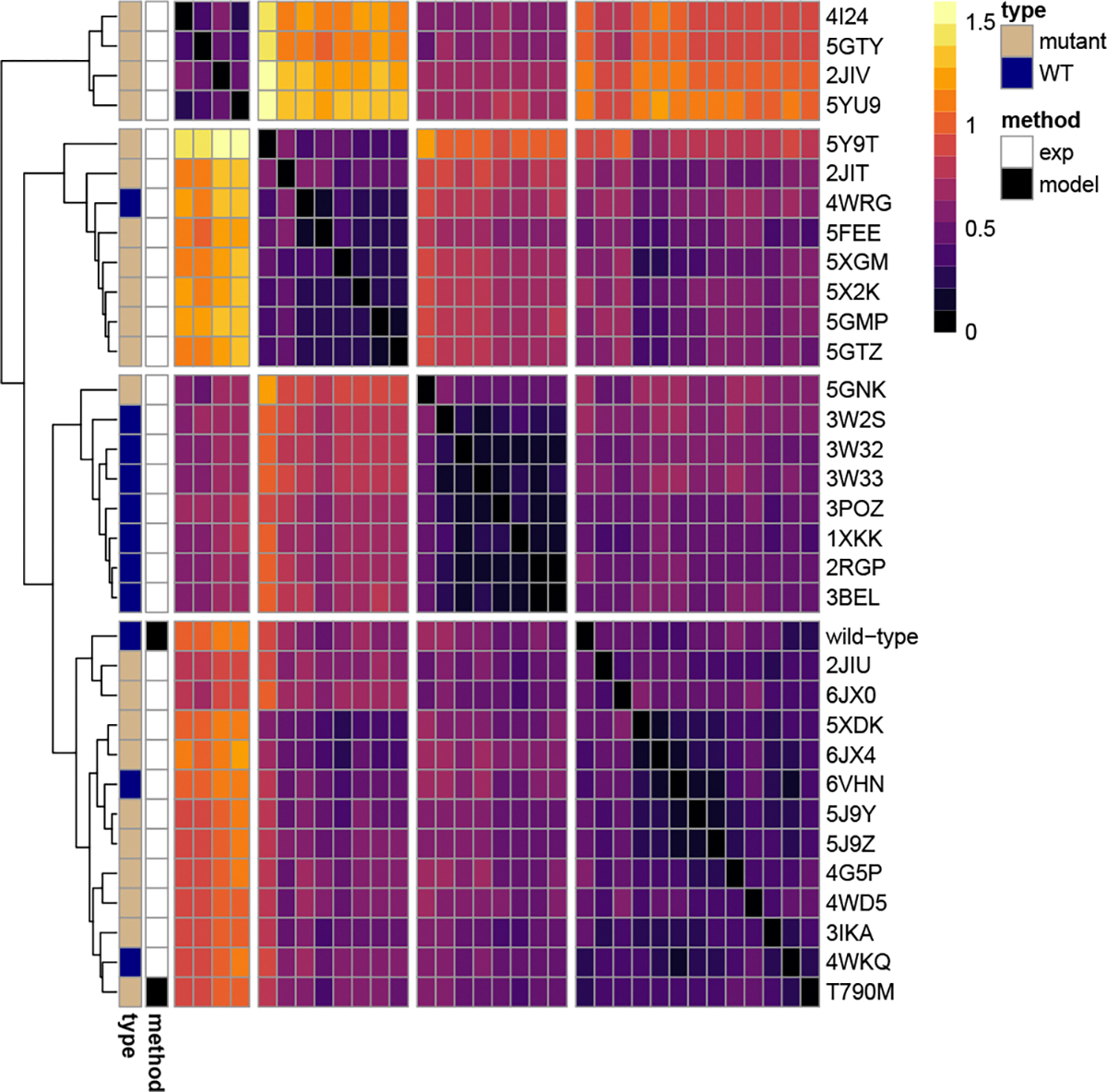
Pairwise RMSD cluster analysis of EGFR wild-type (WT) and mutant models and experimental structures.

To compare the models to experimental structures and determine if the modeling process can predict changes seen by experiments, we did a root mean squared deviation (C*α*RMSD) analysis of the C*α* atoms of the WT and T790M models to experimental structures of the WT and mutant. Figure 2 shows a cluster analysis based on the pairwise RMSD values of all the structures at a 5 Å window surrounding the mutation site. The pairwise RMSD values cluster into four different groups based on similarity. The clusters are separated by white space and can be viewed along the diagonal line of the heatmap. Clusters are numbered from left to right, with Cluster 1 being in the top left of the heatmap and Cluster 4 being in the bottom right of the heatmap. Experimental structures are annotated with white and labeled with their PDB code. Models generated with AlphaFold2 are annotated with black. Annotations are also provided for the amino acid sequence of the structure: WT (navy) or containing the T790M mutation (tan). Metrics on the four clusters are given in Table 1. Size gives the number of structures in the cluster, RMSD is the lowest average RMSD from one structure in the cluster to the rest of the structures in the cluster, and the percent of structures that are either WT or mutant is also given for each cluster. We performed a cluster analysis at a 3-residue and 3 Å window surrounding the mutation site, but the pairwise RMSD values did not form obvious clusters.

### 2.2 Molecular dynamics of predicted mutant protein structures

**Figure 3:**
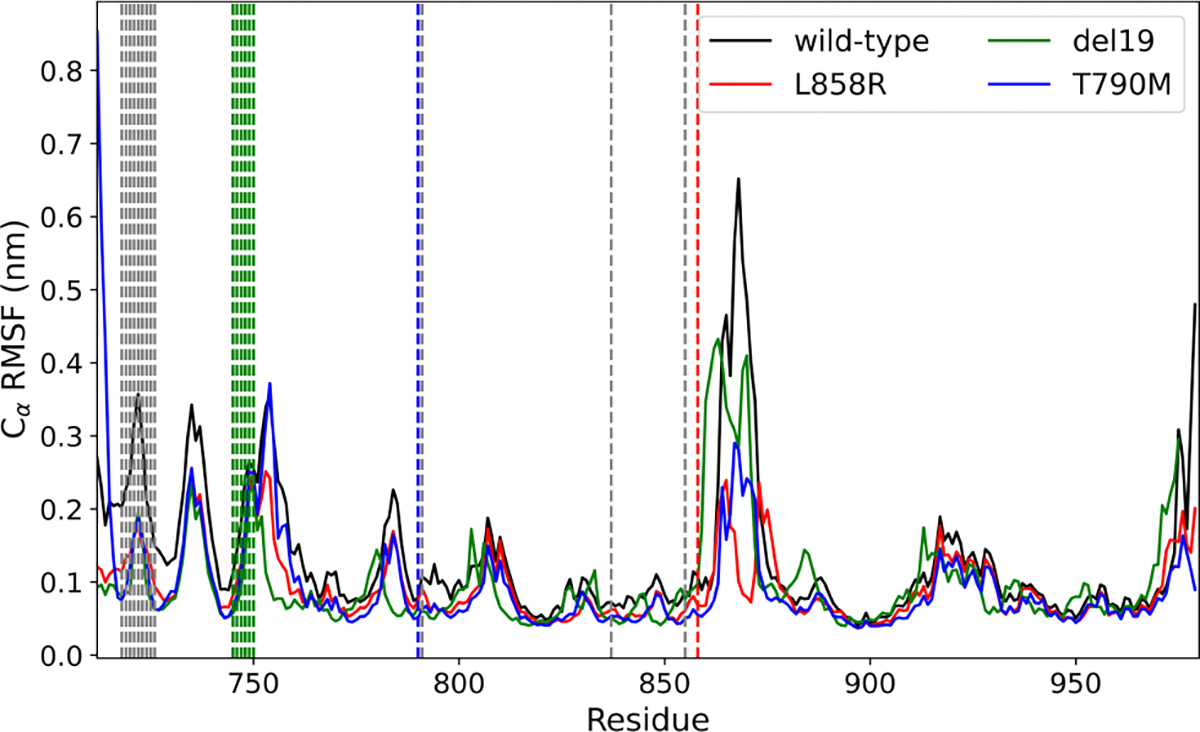
RMSF analysis from simulations of EGFR models of WT and several variants of interest. This shows the flexibility of each residue over the timeline of the simulation, with the higher the RMSF the higher the flexibility. The black dashed vertical lines are binding and active site features from UniProt [1]. Colored vertical lines represent positions of variation.

MD was performed on the predicted WT, T790M, L858R, and del19 structures of EGFR. The trajectories were used for clustering with the GROMOS RMSD-based clustering to select the most distinct frames from the trajectory. The clustering tool works by first doing pairwise RMSD calculations for all frames of the MD trajectory and selecting the largest cluster where all frames are within a given RMSD threshold to the middle frame. These frames are removed, and the process continues until no frames are left. The middle frame is the representative structure for the cluster. Table 2 shows the size (number of frames), conformation (middle frame of the cluster), and the minimum average RMSD from one frame to the rest in each cluster for each protein. A Root Mean Square Fluctuation of C*α* atoms (C*α*RMSF) analysis was also performed and given in Figure 3. An RMSF analysis shows which residues in the protein are most flexible.

### 2.3 Enrichment analysis with ensemble docking

**Figure 4:**
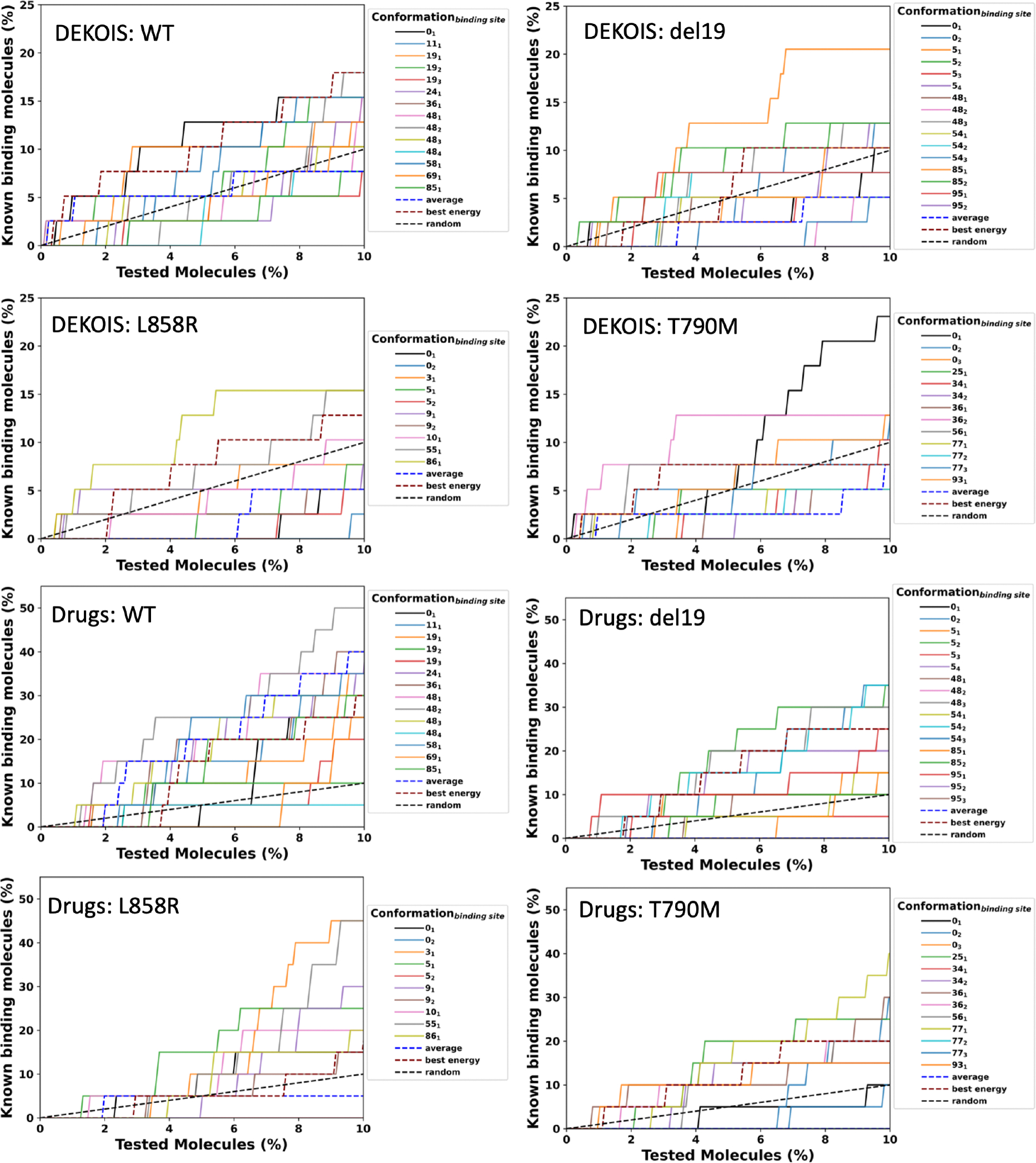
An enrichment analysis showing the percentage of known binding compounds (actives) that can be recovered using binding predictions from a background dataset of small molecules or drugs. Shown here is the DEKOIS 2.0 dataset (top) and the FDA-approved dataset (bottom). Each solid line is a different conformation and/or binding site discovered through MD. The average and best energy lines are the combination of all conformations/binding sites using the average and the best binding energy from all conformations/binding sites. Random gives the number of active compounds that would be expected if selected at random. When the average line is absent, none of the known binding compounds scored in the top 10% using the average score. A compound may score well in some conformations and not well in others.

We performed an enrichment analysis, the percent of known active compounds identified from a benchmark dataset, using an ensemble of protein structures extracted from MD simulations of the AlphaFold2 predicted WT, T790M, L858R, and del19 structures. We used benchmark data from DEKOIS 2.0 [5]. Another analysis uses FDA-approved drugs as a background and compounds interacting with EGFR in DrugBank [29] as the known binding molecules. As shown in Figure 4, the dashed black line in each graph gives the number of actives expected to be identified at random. Each line in a given graph denotes a different conformation or a different binding site in the conformation. The number corresponding to the line is the conformation, with 0 being the starting structure used for MD, which is the output from AlphaFold2 modeling. The increasing value of the number represents the time along the trajectory in which the conformation was generated. Information on the size of the cluster in which the conformation came from can be found in Table 2. The larger the cluster size, the longer the protein is in that conformation. The subscript represents the binding site within the conformation. Therefore, “0_1_” would be the starting structure for MD and the first identified binding site in that conformation using binding site prediction from MOE (Molecular Operating Environment)[27]. In this example, there were 100 total conformations before conformation selection, so “48_4_” is a conformation generated about halfway through the simulation and the fourth identified binding site in that conformation.

### 2.4 Top hits identified through ensemble docking

**Figure 5:**
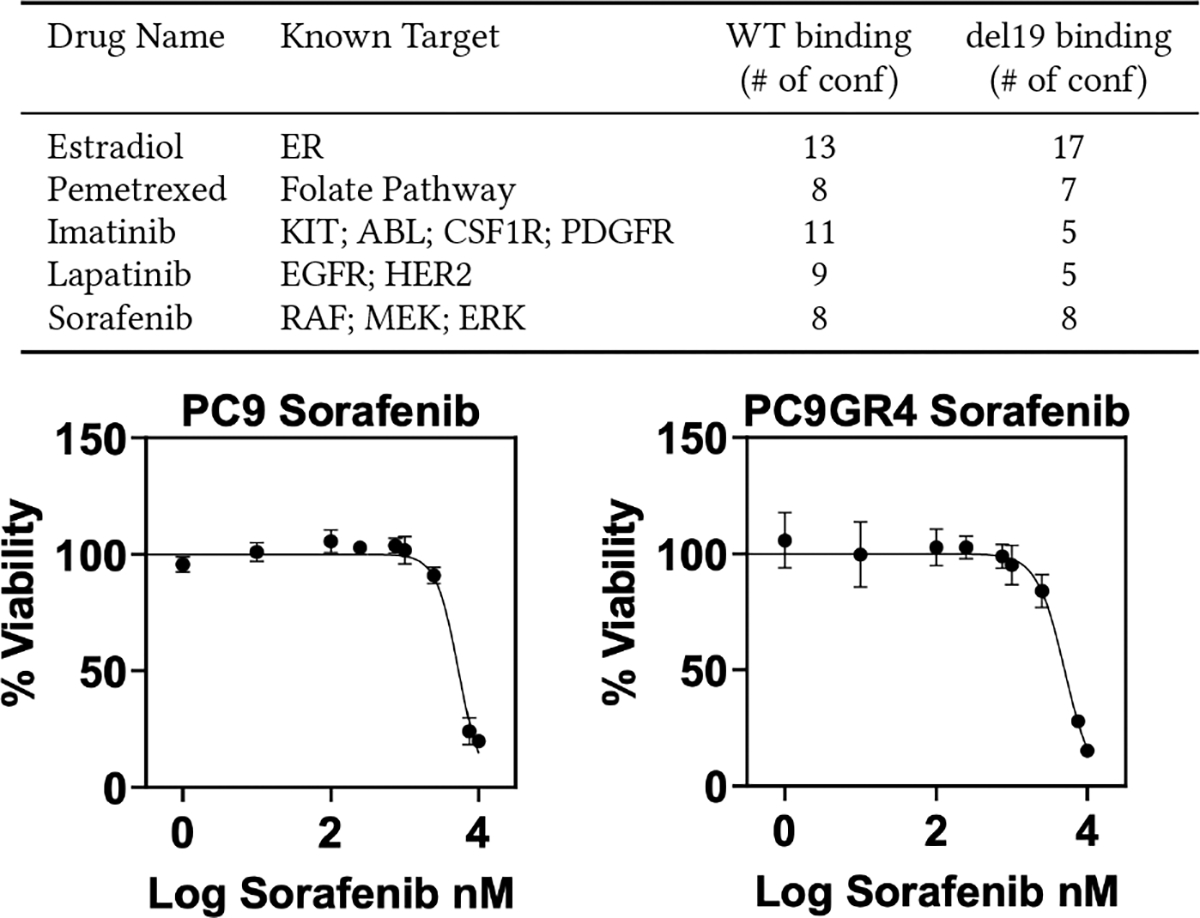
List of selected initial hits and dose response examples.

The selection process to experimentally test compounds was two-tiered. First, lead compounds were selected based on having a high binding score in multiple conformations. This approach utilizes the additional information provided by ensemble docking by selecting compounds that may be more efficacious if they can bind to multiple conformations of the binding site. Next, the compounds were subject to a manual evaluation by the multi-disciplinary team and only compounds with promise for further testing were selected. Initial analysis identified several expected (lapatinib) and unexpected (estradiol) hits, of which sorafenib has anti-growth properties in two EGFR-driven cell lines tested (Figure 5).

### 2.5 Density Functional Theory for more accurate binding predictions

**Figure 6:**
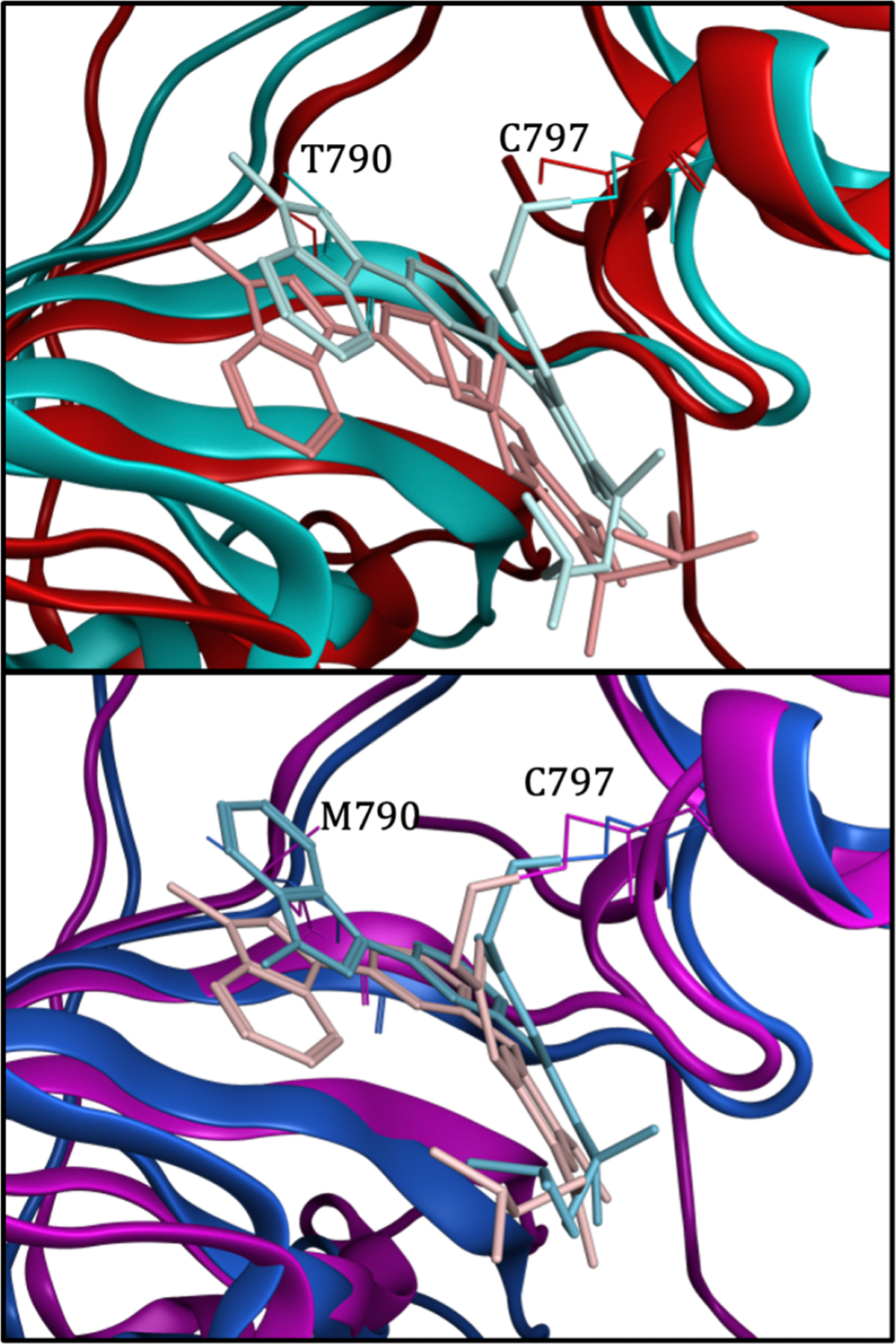
Input for DFT calculations. The top figure shows the binding of osimertinib with the WT structure of EGFR with a covalent bond at C797 in teal (6JXT) and without a covalent bond in red (4ZAU). The bottom figure shows the binding of osimertinib with the T790M structure of EGFR with a flipped binding pose of osimertinib in blue (6JX0) where the drug is closer to the M790 residue. 6JX4 is in pink.

Molecular docking is known to not always be accurate, but its power is in its efficiency and ability to perform enrichment studies using large libraries of chemical compounds. Therefore, we wanted to evaluate more accurate calculations as a complement to experimental validations to better understand the mechanisms of binding. Preliminary work using density functional theory (DFT) in ORCA [17] and a small neighborhood of residues around the bound drug in crystal structures is given in Table 3. Differences in the input structures when we investigate more than one structure per amino acid sequence of the protein are highlighted in Figure 6.

## 3 Discussion

### 3.1 The ability of AlphaFold2 to reproduce structures of known point mutations

Figure 2 shows a cluster analysis of the pairwise RMSD values of our predicted models and experimental structures at a 5 Å window surrounding the mutation site. Not shown here, at a 3-residue and 3 Å window, the mutant and WT models cluster separately, but the clusters are not visually distinct. From 5 Å and up, the mutant and WT models cluster together, but the clusters get more distinct. We can see from Table 1 that Cluster 2 is a predominantly mutant cluster and Cluster 3 is a predominantly WT cluster. Cluster 3, which is mostly WT structures, has the highest similarity, which can be seen by the lowest RMSD value. The smallest cluster, Cluster 1, is the least similar, with the highest RMSD and entirely mutant structures. Also, with a similar RMSD value to Cluster 1, Cluster 4 contains both our models, WT and T790M. Although the models cluster together when looking at a 5 Å window around the mutation site, this cluster is the largest in size with the 3rd largest RMSD value, and the models cluster furthest apart, with the WT model being close to the predominantly WT cluster. These data show the potential of AlphaFold2 to predict the local structural variation caused by mutation. It also demonstrates the importance of performing MD as there are structural changes outside of the 3 Å window that are not picked up by the static modeling.

### 3.2 Insights from molecular dynamics of predicted mutant protein structures

Our preliminary findings with MD (Figure 3) show that there are residue windows with differences in flexibility between WT and mutants in regions outside of modeled mutations, including in known binding sites. This can be seen in the first binding site region in Figure 3 (group of dashed vertical black lines to the far left) and to the right of the L858R mutation (dashed vertical red line).

### 3.3 Enrichment analysis with ensemble docking

A conformation_binding site_ is performing better than random if the corresponding line is above and to the left of the random line, i.e. it is identifying more active compounds than by randomly selecting compounds from the dataset. In Figure 4, for the DEKOIS 2.0 and FDA-approved drug analyses, more conformations are performing better than random at the top 10% of the ranked data in the WT model than any of the mutation models, and T790M has the least. In the DEKOIS analysis, you can see that there is one model that does much better than the rest (the black line), and it comes from conformation 0. This conformation is the direct output from AlphaFold and not a frame from the MD simulation. Since none of the other conformations perform similarly, this shows the importance of running MD simulation, as the predicted single point mutation may not accurately represent the conformational changes far from the mutation site and may not accurately represent the conformational space available to the protein after the mutation occurs. It can also be noted that the best-performing model (yellow line) for the FDA-approved drug analysis comes from conformation 77, a rare conformation based on its size of 1 in Table 2. This means that the known binding drugs may still bind to this mutation but to a conformation that is not as accessible for the mutated protein, significantly reducing the likelihood of binding.

In other preliminary results, H988P which is a neutral mutation, performs like WT, and better than the known mutation causing drug resistance (T790M).

### 3.4 Density Functional Theory for more accurate binding predictions

The binding preference expected for osimertinib based on literature review [30, 31] is T790M > L858R > WT. You can see from the DFT analysis shown in Table 3 that we recovered this trend with 6JX0 > 6JWL > 4ZAU. The conformations that did not follow this trend are also interesting because they give insight into what does not happen. The best score for 6JXT is in WT EGFR. However, in this crystal structure osimertinib made a covalent bond (top of Figure 6 in teal) with C797, and other studies suggest this does not happen in WT [31] and may be a result of the crystallization process. 4ZAU does not have osimertinib covalently bound and may better represent the proper binding conformation. The binding conformation of osimertinib to T790M in 6JX4 (bottom of Figure 6 in pink) has been found in modeling studies, but their work proposed this conformation is incorrect and a result of soaking the crystalized protein with the drug, locking the protein and not allowing conformational changes that happen with binding [30]. They hypothesized what they believe to be the correct conformation based on a simulation in which osimertinib has a flipped conformation in T790M (bottom of Figure 6 in blue) compared to binding with WT or L858R without T790M. And in fact, they recovered that bound conformation by co-crystalizing the protein and osimertinib after an incubation period, the conformation found in 6JX0. This example illustrates the importance of using modeling side-by-side with experimentally derived structures, as neither may tell the whole story.

These results demonstrate the potential to use enrichment analysis with molecular docking to determine mutations of clinical relevance. But will molecular docking reliably predict the binding preferences of any given molecule and different variants of EGFR? In the FDA-approved drug enrichment analysis, osimertinib was not in the top 10% for T790M and del19, but it was in the top 10% for WT and L858R with the same energy value. However, the binding preference of osimertinib favors T790M over WT.

## 4 Materials and Methods

### 4.1 Molecular Modeling

#### 4.1.1 Protein Structure Prediction.

AlphaFold2 directly predicts the 3D protein structure from the amino acid sequence, so the structure of any variant can be predicted. AlphaFold2’s high accuracy comes from a combination of the bioinformatics and physical-based approaches to structure prediction through a novel training procedure utilizing evolutionary, physical, and geometric constraints of protein structure[10]. We refined the conformations of the EGFR WT and mutant models using molecular dynamics (MD) simulations. The starting conformations of the EGFR kinase domain were predicted from the AlphaFold2 webserver. The predicted EGFR structures were compared to experimental structures by calculating the root mean square deviation (RMSD) of the superimposed conformations and generating a pheatmap with clustering using an R script to visually inspect the similarities.

#### 4.1.2 Molecular Dynamics.

Molecular dynamics is a simulation of the physical movement of atoms based on Newton’s equations of motion. The AlphaFold2 predicted structures were used as the initial configurations and surrounded with a 3.0-nm shell of explicit water molecules using the TIP3P[12] model. The simulation system was neutralized using the Na+ and Cl− counterions. The Amber14sb forcefield [15] with OL15 modification was used to describe the bonded and non-bonded interactions in the system as shown in Equation 1.

(1) $$E_{ij}\left(r_{ij}\right)=4\epsilon_{ij}\left({\left(\frac{\sigma_{ij}}{r_{ij}}\right)}^{12}-{\left(\frac{\sigma_{ij}}{r_{ij}}\right)}^{6}\right)+\frac{e_{i}e_{j}}{4\pi \epsilon_{0}r_{ij}}$$

where $E_{ij}$ is the potential energy between atoms i and j due to non-bonded interactions. The variable $r_{ij}$ is the distance between atoms i and $j,\;\epsilon_{ij}$ is the energetic parameter, $\sigma_{ij}$ is the geometric parameter, and $e_{i}$ is the partial charge of atom i.

The simulation process for each system involves three main steps. The first step is energy minimization, conducted using 50,000 steps of the steepest descent method to eliminate close atomic contacts. The second step is an isobaric-isothermal (NPT) ensemble molecular dynamics (MD) simulation, performed for 200 ns under conditions of P = 1 atm and T = 310 K, with an integral step of 2 fs. This step aims to achieve thermodynamic equilibrium and uses the Berendsen method [6] for temperature and pressure control. The third step is a canonical (NVT) ensemble MD simulation, conducted for 500 ns at T = 310 K, with trajectory recording every 200 ps and temperature controlled by the velocity-rescaling method. Interaction parameters include a 1.2 nm cutoff for Lennard-Jones interactions and the particle-mesh Ewald [21] (PME) method for long-range electrostatic interactions. All bonds involving hydrogen atoms are constrained during the simulations. GROMACS 2022.1[ [3] was used for both energy minimization and MD simulations.

#### 4.1.3 Molecular Docking.

We conducted our molecular docking studies using WT, T790M, L858R, and del19 variants of the EGFR kinase domain as targets. The receptor targets were derived from clustered molecular dynamics (MD) simulation representatives. This approach helps account for the inherent flexibility of the protein and sample a wider range of relevant conformations. The receptor targets were prepared for docking and assigned appropriate charges. Two datasets were used for the ligands, the DEKOIS 2.0 [5] benchmark dataset, containing 120 active molecules and 2500 decoy molecules, and the FDA-approved drug dataset from DrugBank [29]. Ligand preparation involved sorting and curating the dataset using RDKit, followed by energy optimization of poor conformers. Subsequently, the ligands were prepared for docking using the AutoDock preparation tools.

Molecular docking simulations were performed using VinaMPI [9, 25]. The MOE 2022 software was used to predict the ligand binding site of the EGFR kinase domain and the docking box was defined using grid boxes with specific dimensions tailored to encompass key predicted binding regions. Separate docking runs were conducted for each EGFR variant—WT, T790M, L858R, and del19—to capture variant-specific interactions. During these simulations, we focused on assessing the free energies of the target-ligand complex.

Post-docking analysis involved evaluating the binding poses and energies to identify potential inhibitors and their binding characteristics. An enrichment analysis was performed to assess the ability of the docking protocol to discriminate between active and decoy compounds in the DEKOIS 2.0 dataset. The percentage of the active molecules found in the top 10% ranked molecules was calculated to evaluate the enrichment performance. The top-scoring compounds from the FDA-approved drug library were further analyzed based on their predicted binding modes, interactions with key residues in the binding site, and estimated binding affinities. Compounds exhibiting favorable binding characteristics and potential for EGFR inhibition were prioritized for subsequent experimental validation and optimization studies.

#### 4.1.4 Quantum Chemical Calculations.

The ORCA 5.0.4. [17] software package was used to perform Density Functional Theory (DFT)- level quantum chemical calculations on osimertinib-bound WT EGFR and its mutants. The preprocessed experimental structures, with PDB IDs 6JTX, 6JX0, 4ZAU, and 6JX4, were used for this study.

Single point energy calculations were conducted for residues within 5 Å of osimertinib. Calculations were done for the apoprotein, the ligand molecule, and the residues-ligand complex using the B3LYP functional and def2-SVP(D4) basis set. The binding energy of the ligand to protein was then calculated using equation 2.

(2) $$E_{\mathrm{binding}}=E_{\mathrm{complex}}-\left[E_{\mathrm{protein}}+E_{\mathrm{ligand}}\right]$$

where $E_{\mathrm{binding}}$ represents the protein-ligand binding energy, $E_{\mathrm{complex}}$ is the energy of the complex molecule, $E_{\mathrm{protein}}$ is the energy of the apoprotein structure, and $E_{\mathrm{ligand}}$ is the energy of the ligand molecule.

#### 4.1.5 Hardware Resources and Running Times.

The computational work in this study was performed on Perlmutter at Berkeley Labs and the Lipscomb Compute Cluster (LCC) at the University of Kentucky. The times in Table 4 are the average time it takes to compute the binding energy for one complex using ORCA, the average time to compute one 500 ns trajectory of MD using GROMACS, and the average time to do one virtual screen for an enrichment analysis (many confirmations of the same protein and one of the chemical libraries – or 16,120 docking calculations). Scripts and result files from this study can be found at https://github.com/imamabi/Insilico-Prediction-EGFR.

### 4.2 Experimental Validation

The team manually reviewed the top-scoring hits from computational binding predictions to assess their suitability for clinical trials if experimental validation is successful. For selected compounds, 10-point dose-response assays with drug doses surrounding the IC50 concentration of the drug were performed in experimental replicates using CellTiter-Glo in 96-well formats. PC9 and PC9GR4 (add Ercan 2010, PMC2859699) were plated at 2,500 cells per well and fed drug the following day. At 96 hours, CellTiter-Glo was added and luminescence in each well was measured. Luminescence relative to the vehicle control wells was calculated for each drug dose, and non-linear regressions using log(inhibitor) vs response were generated using GraphPad Prism software with top constrained to 100% and bottom constrained to 0%. IC50 values for the drugs in cell lines harboring EGFR mutations of interest and control cell lines were extrapolated.

## 5 Conclusions

This preliminary work has included the creation of a baseline workflow in which we can exchange computational components and a preliminary study using known and benchmark data. We have found that computationally efficient molecular docking calculations provide insights into drug resistance when using large libraries of compounds with a subset of known binding compounds to the WT protein. We noted that these calculations may not be accurate enough to dependably rank an individual compound correctly for its binding preference to different mutants. We found that quantum chemistry tools are powerful enough to do this but require very accurate starting structures, which may be beyond current computational capabilities and still need experimental validation. The use of modeling in conjunction with complementary methods like conformational search, docking, and binding evaluation allows for better functional interpretation of mutations. In the future, we expect AlphaFold3 to expand the range of therapeutic targets amendable to this overall approach to include protein complexes involving nucleic acids, modified residues, and/or small molecules.

## Figures and Tables

**Table 1:** Cluster metrics from pairwise RMSD cluster analysis. Clusters can be viewed from left to right along the diagonal of the heatmap of Figure 2. Size is the number of structures in the cluster. RMSD is the similarity of all structures in the cluster. The percent of structures in each cluster, which are WT or mutant, is also given.

Cluster	Size	RMSD	%WT	%Mutant

1	4	.295	0	100
2	8	.259	12.5	87.5
3	8	.184	87.5	12.5
4	13	.292	23.1	76.9

**Table 2:** Conformations extracted from MD using RMSD-based clustering. Size is the number of frames in the cluster, conformation is the representative frame for the cluster, and RMSD is the similarity of all frames in a cluster. The conformation number relates to the time along the trajectory in which the frame was produced.

Cluster #	WT			T790M			L858R			del19		
	Size	Conf	RMSD	Size	Conf	RMSD	Size	Conf	RMSD	Size	Conf	RMSD

1	66	36	.102	82	25	.066	89	86	.065	83	95	.099
2	12	24	.091	14	56	.071	6	3	.082	7	85	.099
3	10	58	.108	2	34	.116	2	5	.095	6	54	.108
4	8	85	.088	1	36	0	2	55	.137	4	5	.101
5	2	48	.124	1	93	0	1	10	0	1	48	0
6	1	69	0	1	77	0	1	9	0			
7	1	19	0									
8	1	11	0									

**Table 3:** Results of binding predictions using DFT and 5 Å binding pocket. Calculations were done using crystal structures described here. The more negative the binding energy the better the prediction. Expected order of binding is T790M > L858R > WT.

PDB ID	Binding Energy (kcal/mol)	EGFR	Note

6JXT[30]	−577.57	WT	covalent bond with osimertinib
6JX0[30]	−534.45	T790M	protein co-crystalized with osimertinib
6JWL[30]	−499.87	L858R	protein crystal soaked with osimertinib
4ZAU[31]	−409.33	WT	no covalent bond with osimertinib
6JX4[30]	73.05	T790M	protein crystal soaked with osimertinib

**Table 4:** Hardware and runtimes

Computer	CPU (GPU)	MPI processes	Software	Time (hr)

Perlmutter	AMD 7763	32–48	ORCA	20.31
LCC	Intel 6230 (V100)	10	GROMACS	98.25
LCC	Intel 6252	48	VinaMPI	7.43
